# COVID-19 Mortality Update — United States, 2022

**DOI:** 10.15585/mmwr.mm7218a4

**Published:** 2023-05-05

**Authors:** Farida B. Ahmad, Jodi A. Cisewski, Jiaquan Xu, Robert N. Anderson

**Affiliations:** 1 National Center for Health Statistics, CDC.

The National Center for Health Statistics’ (NCHS) National Vital Statistics System (NVSS) collects and reports annual mortality statistics using U.S. death certificate data. Provisional data, which are based on the current flow of death certificate data to NCHS, provide an early estimate of deaths before the release of final data.* This report summarizes provisional U.S. COVID-19 death data for 2022. In 2022, COVID-19 was the underlying (primary) or contributing cause in the chain of events leading to 244,986 deaths^†^ that occurred in the United States. During 2021–2022, the estimated age-adjusted COVID-19–associated death rate decreased 47%, from 115.6 to 61.3 per 100,000 persons. COVID-19 death rates were highest among persons aged ≥85 years, non-Hispanic American Indian or Alaska Native (AI/AN) populations, and males. In 76% of deaths with COVID-19 listed on the death certificate, COVID-19 was listed as the underlying cause of death. In the remaining 24% of COVID-19 deaths, COVID-19 was a contributing cause. As in 2020 and 2021, during 2022, the most common location of COVID-19 deaths was a hospital inpatient setting (59%). However, an increasing percentage occurred in the decedent’s home (15%), or a nursing home or long-term care facility (14%).^§^ Provisional COVID-19 death estimates provide an early indication of shifts in mortality trends and can help guide public health policies and interventions aimed at reducing COVID-19–associated mortality.

This report analyzed provisional NVSS death certificate data for deaths among U.S. residents within the United States during January–December 2022. COVID-19–associated death counts and rates include deaths for which COVID-19 was listed on the death certificate as an underlying or contributing cause of death. NCHS tabulated the number and rates of COVID-19 deaths by age, sex, and race and ethnicity (categorized as AI/AN, non-Hispanic Asian [Asian], non-Hispanic Black [Black], non-Hispanic Hawaiian or other Pacific Islander, non-Hispanic White [White], Hispanic or Latino [Hispanic], non-Hispanic persons of more than one race [multiracial], and unknown), and U.S. Department of Health and Human Services (HHS) region of residence. NVSS data in this report exclude deaths among residents of U.S. territories and foreign countries. This activity was reviewed by CDC and was conducted consistent with applicable federal law and CDC policy.^¶^

NCHS coded the causes of death according to the *International Classification of Diseases, Tenth Revision*, which details disease classification and the designation of underlying cause of death (*1*,*2*). The underlying cause of death is the disease or injury that initiated the chain of morbid events leading directly to death. Contributing causes are conditions that are also part of the chain of events leading to death. Among all deaths with COVID-19 listed on the death certificate, the leading causes of death were ranked by number of deaths per underlying cause of death (*3*). Race and ethnicity were unknown for 679 (0.28%) decedents and age was unknown for four (<0.01%). Age-adjusted death rates were calculated by sex, race and ethnicity, and residence. Crude death rates were calculated by age. The population data used to calculate deaths rates are July 1, 2021 estimates, based on the Blended Base produced by the U.S. Census Bureau (*4*,*5*). Place of death is noted on the death certificate and is determined by both the location where the death was pronounced and the physical location where the death occurred (*6*). In this report, place of death is categorized as decedent's home, hospice facility, hospital inpatient setting, hospital outpatient or emergency department, nursing home or long-term care facility, or other (which includes being dead on arrival, other, and unknown).

In 2022, COVID-19 was listed as an underlying or contributing cause of 244,986 (61.3 per 100,000) deaths (Table 1). COVID-19–associated death rates were lowest among children and adolescents aged 5–14 years (0.5) and highest among adults aged ≥85 years (1,224.2). In 2022, similar to previous years, the age-adjusted COVID-19–associated death rate for males (76.3) was higher than that for females (49.8). Age-adjusted COVID-19 death rates were lowest in multiracial (26.7) and Asian persons (34.1) and highest in AI/AN persons (86.8).

**TABLE 1:** Provisional* number and rate^†^ of COVID-19–associated^§^ deaths, by demographic characteristic and U.S. Department of Health and Human Services region^¶^ of residence **—** National Vital Statistics System, United States, 2020–2022

Characteristic	No. of deaths (rate)^†^		
	2020	2021	2022
**Total**	**384,536 (93.2)**	**462,193 (115.6)**	**244,986 (61.3)**
**Age group, yrs**			
<1	52 (1.4)	167 (4.7)	231 (6.5)
1–4	25 (0.2)	66 (0.4)	152 (1.0)
5–14	68 (0.2)	185 (0.4)	203 (0.5)
15–24	612 (1.4)	1,652 (3.8)	641 (1.5)
25–34	2,609 (5.7)	7,033 (15.5)	2,376 (5.2)
35–44	6,756 (16.0)	17,412 (40.1)	5,183 (11.9)
45–54	18,250 (45.2)	39,360 (96.7)	12,169 (29.9)
55–64	45,377 (107.0)	79,199 (185.0)	30,526 (71.3)
65–74	82,055 (252.1)	111,412 (330.9)	53,228 (158.1)
75–84	106,020 (644.4)	110,536 (682.1)	67,116 (414.1)
≥85	122,707 (1,842.9)	95,168 (1,592.6)	73,157 (1,224.2)
Unknown	5 (—)	3 (—)	4 (—)
**Sex**			
Female	175,818 (73.9)	202,687 (91.8)	112,287 (49.8)
Male	208,718 (117.0)	259,506 (144.5)	132,699 (76.3)
**Race and ethnicity**			
AI/AN, NH	4,615 (190.8)	5,053 (201.8)	2,115 (86.8)
Asian, NH	13,523 (67.2)	13,707 (66.6)	6,786 (34.1)
Black or African American, NH	61,401 (154.8)	61,959 (151.4)	28,695 (72.9)
NH/OPI, NH	691 (123.5)	1,175 (200.9)	378 (67.8)
White, NH	232,555 (74.1)	304,586 (105.0)	180,212 (61.2)
Hispanic	69,069 (164.8)	72,910 (161.7)	25,076 (60.9)
Multiracial, NH	1,141 (31.9)	2,018 (50.7)	1,045 (26.7)
Unknown	1,541 (—)	785 (—)	679 (—)
**HHS region^¶^**			
1	19,725 (94.3)	12,791 (64.1)	9,877 (49.5)
2	56,707 (151.6)	33,778 (90.0)	21,578 (57.4)
3	34,918 (84.7)	42,482 (107.0)	25,722 (64.9)
4	70,574 (78.7)	117,619 (137.4)	56,695 (65.5)
5	66,484 (97.9)	69,627 (106.6)	42,540 (65.1)
6	52,638 (114.0)	72,359 (158.6)	30,847 (69.3)
7	18,521 (99.1)	18,063 (102.7)	11,287 (63.7)
8	11,281 (84.6)	13,102 (98.5)	6,759 (52.2)
9	46,549 (76.3)	68,415 (115.4)	31,262 (53.0)
10	7,139 (41.5)	13,957 (82.4)	8,419 (50.9)

**Abbreviations:** AI/AN = American Indian or Alaska Native; HHS = U.S. Department of Health and Human Services; NH = non-Hispanic; NH/OPI = Native Hawaiian or other Pacific Islander.

* National Vital Statistics System provisional data for 2022 are incomplete. Data from December 2022 are less complete because of reporting lags. Data for 2020 and 2021 are final. These data exclude deaths that occurred in the United States among residents of U.S. territories and foreign countries.

^†^ Deaths per 100,000 standard population. Age-adjusted death rates are provided overall and by sex, race and ethnicity, and HHS region.

^§^ Deaths with COVID-19 listed as an underlying or contributing cause of death, with *International Classification of Diseases, Tenth Revision* (code U07.1).

^¶^ https://www.hhs.gov/about/agencies/iea/regional-offices/index.html

The COVID-19–associated age-adjusted death rate was lowest (49.5 per 100,000) in HHS Region 1 (Connecticut, Maine, Massachusetts, New Hampshire, Rhode Island, and Vermont) and highest (69.3) in Region 6 (Arkansas, Louisiana, New Mexico, Oklahoma, and Texas). The second highest age-adjusted COVID-19 death rate (65.5) and highest number of COVID-19–associated deaths (56,695) occurred in Region 4 (Alabama, Florida, Georgia, Kentucky, Mississippi, North Carolina, South Carolina, and Tennessee).

For surveillance purposes, and in this report, COVID-19-associated deaths are deaths with COVID-19 as an underlying or contributing cause of death. In 2022, among deaths with COVID-19 listed on the death certificate, COVID-19 was listed as the underlying cause in 76% of deaths (Table 2). For deaths with COVID-19 listed as a contributing cause, heart disease and cancer were the next most frequent underlying causes, listed in 6% and 4% of deaths, respectively. Other underlying causes of death for COVID-19–associated deaths included chronic lower respiratory diseases, stroke, Alzheimer disease, diabetes, unintentional injuries, kidney disease, and Parkinson disease. COVID-19 was listed as the sole cause of death on 3.6% of death records in 2022, compared with 5.6% in both 2020 and 2021. In 2022, COVID-19 deaths that listed additional causes of death included an average of 4.5 listed contributing causes, compared with 4.2 in 2021 and 4.0 in 2020.

**TABLE 2:** Provisional* leading underlying causes of death with corresponding *International Classification of Diseases, Tenth Revision* codes and number of deaths for COVID-19–associated deaths**^†^**** —** National Vital Statistics System, United States, 2022

Rank	Underlying cause of death (ICD-10 code)	No. (%)
1	COVID-19 (U07.1)	186,702 (76.2)
2	Heart disease (I00-I09, I11, I13, and I20–I51)	14,415 (5.9)
3	Cancer (C00-C97)	9,579 (3.9)
4	Chronic lower respiratory diseases (J40–J47)	3,976 (1.6)
5	Stroke (I60–I69)	3,881 (1.6)
6	Alzheimer disease (G30)	3,112 (1.3)
7	Unintentional injuries (V01–X59 and Y85–Y86)	2,572 (1.0)
8	Diabetes mellitus (E10–E14)	2,399 (1.0)
9	Kidney disease (N00–N07, N17–N19, and N25–N27)	1,259 (0.5)
10	Parkinson disease (G20–G21)	987 (0.4)

**Abbreviation**: ICD-10 = *International Classification of Diseases, Tenth Revision*.

* National Vital Statistics System provisional data for 2022 are incomplete. Data from December 2022 are less complete because of reporting lags. These data exclude deaths that occurred in the United States among residents of U.S. territories and foreign countries.

^†^ Deaths with COVID-19 listed as an underlying or contributing cause of death, with ICD-10 (code U07.1).

In 2022, approximately 59% of COVID-19–associated deaths occurred in a hospital inpatient setting, followed by 15% in the decedent’s home, and 14% in a nursing home or long-term care facility. The three most common places of death for COVID-19 deaths have remained similar since the beginning of the pandemic, but the proportion of deaths occurring in each setting has shifted over time (Figure). The percentage of deaths occurring in a hospital inpatient setting decreased from nearly 70% during 2020–2021 to approximately 60% in 2022.

**FIGURE:**
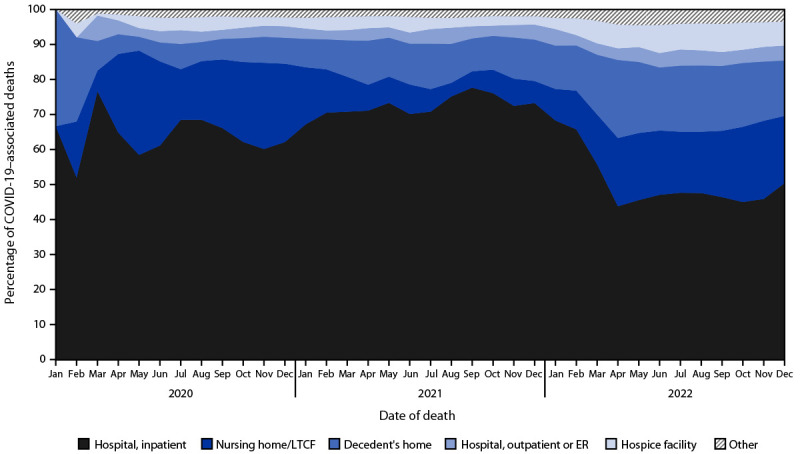
Percentage* of COVID-19–associated deaths,^†^ by location of death — National Vital Statistics System, United States, January 2020–December 2022. **Abbreviations**: ER = emergency room; LTCF = long-term care facility. * Percentages are provisional. National Vital Statistics System provisional data for 2022 are incomplete, and data from December 2022 are less complete because of reporting lags. Data for 2020 and 2021 are final. These data include deaths that occurred in the United States among residents of U.S. territories and foreign countries. ^†^ Deaths with COVID-19 listed as an underlying or contributing cause of death, with *International Classification of Diseases, Tenth Revision* (code U07.1).

## Discussion

During January–December 2022, 244,986 deaths with COVID-19 listed as an underlying or contributing cause of death occurred among U.S. residents. The age-adjusted COVID-19 death rate was 61.3 per 100,000 persons. COVID-19–associated death rates were highest among males, older adults, and AI/AN persons. The COVID-19–associated age-adjusted death rate varied by HHS region, with the lowest rates in New England (Region 1) and highest rates in the south central United States (Region 6).

Some demographic characteristics of COVID-19–associated deaths have remained similar since 2020; however, changes in other characteristics have occurred over time. During the first 2 years of the COVID-19 pandemic, for example, COVID-19 was listed as the underlying cause on approximately 90% of death certificates. In 2022, the percentage of deaths with COVID-19 as the underlying cause decreased to 76% (*7*). Changes were also observed in the setting where COVID-19 deaths are occurring. Whereas most COVID-19 deaths still occur in hospital inpatient settings, the proportion of those deaths decreased in 2022, as more deaths occurred in decedents’ homes and nursing homes or long-term care facilities.

The findings in this report are subject to at least three limitations. First, data are provisional, and numbers and rates might change as additional information is received. Described changes in mortality trends might be underestimates. Second, timeliness of death certificate submission can vary by jurisdiction. As a result, the national or regional distribution of deaths might be affected by the distribution of deaths reported from jurisdictions reporting later, which might differ from those in the United States or in a region overall. Finally, potential exists for misclassification of certain categories of race (i.e., AI/AN and Asian) and Hispanic ethnicity reported on death certificates (*8*). Thus, death rates for some groups might be under- or overestimated.

This report provides an overview of COVID-19–associated mortality in the United States in 2022 and highlights changes in the characteristics of COVID-19 deaths. These data provide updated information that advances understanding of the impacts of COVID-19 on mortality and how these have continued to shift during the course of the pandemic. These findings also help to guide public health policies and interventions intended to reduce severe COVID-19 impact by providing insight into groups that remain vulnerable to COVID-19–associated mortality.

**Summary**

**What is already known about this topic?**

COVID-19 was associated with approximately 244,000 deaths in the United States during January–December 2022.

**What is added by this report?**

The age-adjusted COVID-19 death rate declined 47% compared with 2021. As in 2020 and 2021, most COVID-19 deaths occurred in a hospital inpatient setting, but an increasing percentage occurred in the decedent’s home or a nursing home or long-term care facility.

**What are the implications for public health practice?**

Provisional death estimates provide an early signal about shifts in COVID-19 mortality trends. Timely and actionable data can guide public health policies and interventions for populations experiencing higher COVID-19–associated mortality.

## Footnotes

* https://wonder.cdc.gov; https://www.cdc.gov/nchs/nvss/deaths.htm

† Based on death records received and processed as of April 2, 2023, for deaths occurring in the United States among U.S. residents. Data included in this analysis include >99% of deaths occurring in 2022.

§
https://data.cdc.gov/NCHS/Provisional-COVID-19-Deaths-by-Place-of-Death-and-/4va6-ph5s

¶ 45 C.F.R. part 46, 21 C.F.R. part 56; 42 U.S.C. Sect. 241(d); 5 U.S.C. Sect. 552a; 44 U.S.C. Sect. 3501 et seq.
